# Optimizing molecular weight of octyl chitosan as drug carrier for improving tumor therapeutic efficacy

**DOI:** 10.18632/oncotarget.19452

**Published:** 2017-07-22

**Authors:** Min Zhang, Yi Ma, Zhaohui Wang, Zhihao Han, Weidong Gao, Yueqing Gu

**Affiliations:** ^1^ State Key Laboratory of Natural Medicines, Jiangsu Key Laboratory of Drug Screening, Department of Biomedical Engineering, School of Engineering, China Pharmaceutical University, Nanjing 210009, China

**Keywords:** molecular weight, octyl chitosan, drug delivery, therapeutic efficacy improvement, reduced side effect

## Abstract

Macromolecular drug carriers have attracted much attention taking advantage of passive tumor targeting property and excellent biocompatibility. For many biomedical applications, however, the effectiveness of the carriers is insufficient, which complicate further development into clinical use. Here, we systematically investigated the effects of molecular weight (from 1KDa to 300KDa) of macromolecular drug carrier, octyl chitosan, on tumor accumulation and penetration, as well as drug loading and releasing profiles. It was found that the molecular weight of chitosan influenced the cellular uptake and pharmacokinetic behavior of the nanocarriers, which ultimately determined their drug delivery efficiency. Interestingly, increased molecular weight of chitosan decreased its cellular uptake but increased its resident time in blood, which provided ample time for tumor accumulation. Moreover, the molecular weight altered the drug loading capability and release profile. Our results demonstrated that 10KDa octyl chitosan was an ideal candidate for anticancer drug delivery, which could deliver anticancer agent to tumor tissues and release drugs in tumor cells more effectively than those of other molecular weights, and finally result in better therapeutic effect.

## INTRODUCTION

The emergence of nanoparticulate pharmaceutical carriers have enhanced the therapeutic effects of many anticancer therapeutic agents both in fundamental research and clinical setting [1–6]. Encapsulation in nanocarriers protects drugs against degradation [7], prolongs their circulation in blood [8], increases tumor accumulation [9] and reduces side effects [10]. These advantages have facilitated the development of the nanocarriers in various fields of biomedical research. However, several obstacles still exist, hindering further clinical application. Many nanocarriers have failed to fulfill the expected drug delivery efficacy [11–13]. Effective drug delivery is determined by multifaceted factors among which the drug accumulation and release at tumor site *in vivo* are particularly important [14, 15]. Therefore, improvement of tumor accumulation and drug release property of nanocarriers would enhance drug availability and, as a result, therapeutic efficiency.

Macromolecular drug carriers, such as macromolecular micelles, accumulate in tumor tissues by either passive targeting through enhanced permeability and retention (EPR) effect or active targeting via specific affinity [16]. In addition to enhancing the concertation of nanodrug in tumors and improving the therapeutic efficacy, two sets of parameters should be considered- one set that increases the accumulation of the nanocarriers in tumors, such as perfusion, vascular permeability, circulation time, and tumor-specific binding, and the other set that limits tumor localization, such as clearance through a vascular or lymphatic route [17, 18]. For passively targeted nanocarriers, only permeability, circulation time, and clearance are variable. These variables mostly depend on the molecular weight and charge of the drug carrier. In this study, we focused on the influence of the molecular weight of macromolecules. Additionally, nanocarriers usually show unsatisfactory drug-release profile [19], for example, premature drug release in circulation or slow diffusion lasting for many days. Both of the two drug release profiles hinder optimal drug availability inside tumor cells. The molecular weight of nanocarriers could affect physical property of the nanocarriers, which might influence the drug release profile, and further affect therapeutic efficacy. Herein, we thoroughly studied the influence of the molecular weight of macromolecule chitosan on tumor accumulation and drug release property.

Chitosan is a biocompatible and biodegradable natural polysaccharide [20], and it is increasingly used in drug delivery [21], tissue engineering [22], and wound healing [23]. The positive charge of chitosan makes chitosan-based nanocarriers bind more efficiently to mammalian cells [24]. In this research, a series of chitosan nanocarriers were prepared by modified octyl to chitosan (1-300KDa) as the hydrophobic group (octyl chitosan, OC). The passive targeting ability and tumor penetration of these nanocarriers was evaluated. We then measured the drug loading capacity and release profile of OC with different molecular weights of chitosan. Finally, the therapeutic efficacy of drug loaded nanocarriers were specifically explored, revealing 10KDa as the optimal molecular weight for drug delivery over the 1-300KDa range. Moreover, the toxicity of the nanocarriers with different molecular weight was further investigated. Through these systematic studies, it is of interest to demonstrate that molecular weight has tremendous impacts on drug delivery, which will allow for an optimized approach to deliver drugs more safely and effectively.

## RESULTS AND DISCUSSION

### Characterization of OC with different molecular weights

**Figure 1:**
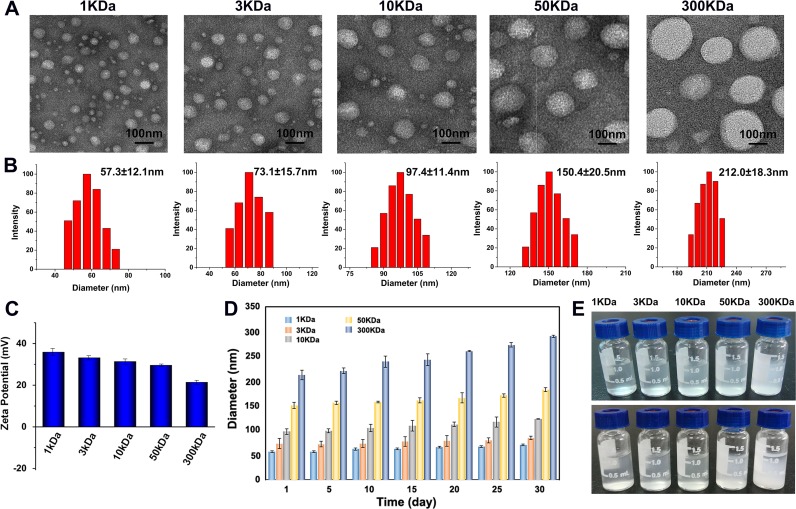
Characterization of 1, 3, 10, 50, 300 KDa OC **(A)** TEM images and the corresponding size distribution histograms **(B)** of the OC with different molecular weights. **(C)** Zeta Potential of as-prepared OC. **(D)** The size distribution histograms of different molecular weight OC after 30 days’ storage. **(E)** The photograph of as-prepared OC before (up) and after (down) 30 days’ storage.

In order to investigate the influence of molecular weight on drug delivery, octyl-chitosan (OC) with molecular weight ranging from 1 to 300KDa was synthesized in this study. TEM images displayed that the as-prepared nanocarriers have nearly spherical morphology (Figure 1A) and the hydrodynamic diameters of OC were measured by dynamic light scattering (DLS). The sizes of OC were found to be about 57.3±12.1, 73.1±15.7, 97.4±11.4, 150.4±20.5, and 212.0±18.3 nm (Figure 1B), which increased in proportion to the molecular weight of chitosan. Zeta potential is used to evaluate or predict the physical stability of the particle disperse system. High absolute value of Zeta potential generally indicates larger electrostatic repulsion force among particles and better physical stability of the system. The Zeta-potentials of each molecular weight of OC were detected to be 35.86, 33.14, 31.37, 29.54, and 21.37 mV respectively, which gradually decreased with increased molecular weight (Figure 1C). The decreased charge might lead to less stability of OC. As displayed in Figure 1D and 1E, the particle size of lower molecular weight nanocarriers was almost not changed during 30 days’ storage, while 300KDa OC showed aggregation. To further investigate the dispersion of OC in biological environments, additional stability experiments were conducted in different media. As shown in Supplementary Figure 1A, all kinds of OC with different molecular weights were well dispersed in the relevant media in a discrete state.

The cytotoxicity of different molecular weight nanocarriers was then examined and compared using normal cell lines L02 (liver cells) and HUVEC (human umbilical vein endothelial cells) by MTT assay. The nanocarriers with different molecular weights showed no significant inhibition in the both cell lines, though the highest concentration of 2 mg/mL caused slight decrease of cell viability (Supplementary Figure 2A and 2B). Furthermore, the breast cancer cell line MCF7 was used to evaluate the toxicity of OC. As shown in Supplementary Figure 3, the OC with different molecular weights demonstrated no significant inhibition in MCF7 cells, confirming the good biocompatibility of OC nanocarriers. The *in vivo* toxicity of these nanocarriers was further tested on mice. The blood routine and biochemical parameters were determined after intravenous injection of OC for 30 days, at a 5-time dose of normal administration. Compared to the saline control, parameter changes induced by the nanocarriers of different molecular weights were within the normal range, although Creatine kinase (CK) value obviously elevated with increased molecular weight (Supplementary Figure 2C and 2D). Clinically, creatine kinase is a marker of damage of CK-rich tissue such as in heart, muscle, brain and so on. The elevated CK value was might caused by cardiac injury, but no obvious pathological changes were found in tissues (Supplementary Figure 4). Both body weights and tissue index showed no significantly toxicity during 30 days’ treatment (Supplementary Figure 2E and 2F).

### Cellular uptake of OC with different molecular weights

**Figure 2:**
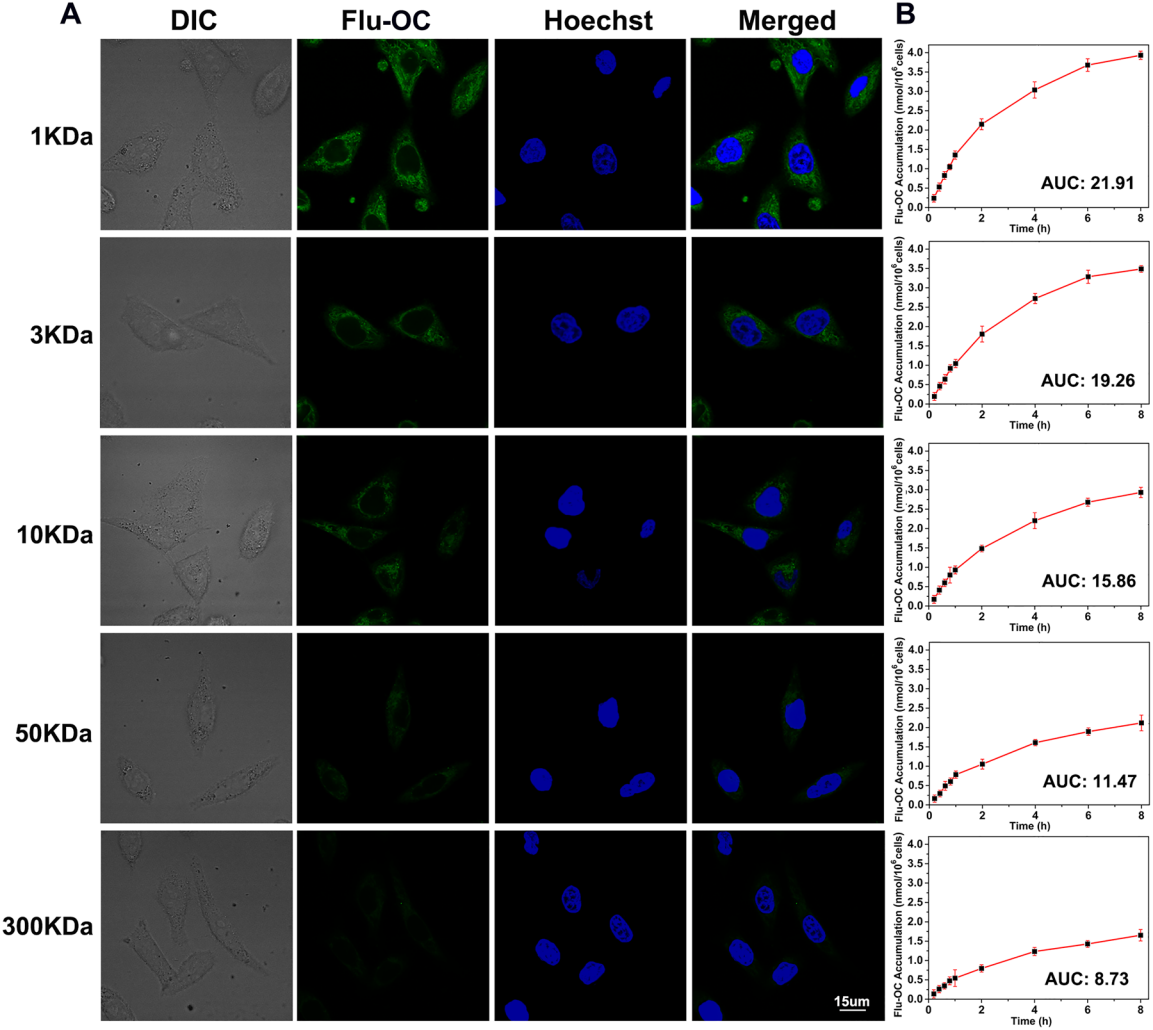
Intracellular uptake of fluorescein labelled OC (Flu-OC) with different molecular weights in MCF7 cells **(A)** Laser confocal fluorescence images of MCF7 cells after incubation with Flu-OC (green) of different molecular weights for 4 h. Nuclei were stained by Hoechst 33342 (blue). **(B)** Mean concentration-time profiles of Flu-OC in MCF7 cells measured by UV-Vis spectrophotometer after incubation for different time (0.2 h, 0.4 h, 0.6 h, 0.8 h, 1 h, 2 h, 4 h, 6 h, 8 h).

To compare the cellular uptake of OC with different molecular weights, fluorescein labeled nanocarriers (Flu-OC) were incubated with MCF7 breast carcinoma cells. As exhibited in Figure 2, all of the micelles were distributed in the cytoplasm with good uniformity. Pale green fluorescence signal was observed in larger molecular weight OC (50KDa and 300KDa) treated MCF7 cells, while other three smaller molecular weight OC displayed stronger signal at the same time, among which 1KDa OC with the smallest particle size showed the strongest fluorescence signal. Additionally, the quantification of the fluorescence was demonstrated by the area under the Flu-OC concentration–time curve (AUC) of each group. The intracellular concentration of Flu-OC was decreased with increased molecular weight, and the AUC of 1KDa Flu-OC was nearly three times of that of 300KDa Flu-OC. Therefore, larger molecular weight of the nanocarriers might decrease the intracellular uptake of the nanoparticles.

To investigate the mechanism of cellular uptake of OC with different molecular weights, the inhibition experiment was conducted. Flu-OC of different molecular weights was incubated with MCF7 cells pre-treated with β-cyclodextrin (inhibitor of caveolin) treated and sucrose (inhibitor of clathrin), respectively. As shown in Supplementary Figure 5, the treatment of β-cyclodextrin significantly reduced the cellular uptake of 1 and 3 KDa OC, slightly decreased the uptake of 10 KDa OC, but barely undermined the uptake of 50 and 300 KDa OC, indicating the caveolin-mediated endocytosis of small molecular weight nanocarriers (1, 3 and 10 KDa). In contrast, the fluorescence imaging of sucrose treated MCF7 cells implied the clathrin-mediated endocytosis of large molecular weight nanocarriers (10, 50 and 300 KDa). The results were in consist with previously reports [25, 26]. Moreover, the positive charges of OC could lead to the absorption to the surface of cells, which mostly shows negative charges. As the molecular weight increased, the positive charge of OC decreased (Figure 1C), leading to the lower trend of absorption and further lower uptake efficiency by the MCF7 cells.

### *In vivo* distribution of OC with different molecular weights

**Figure 3:**
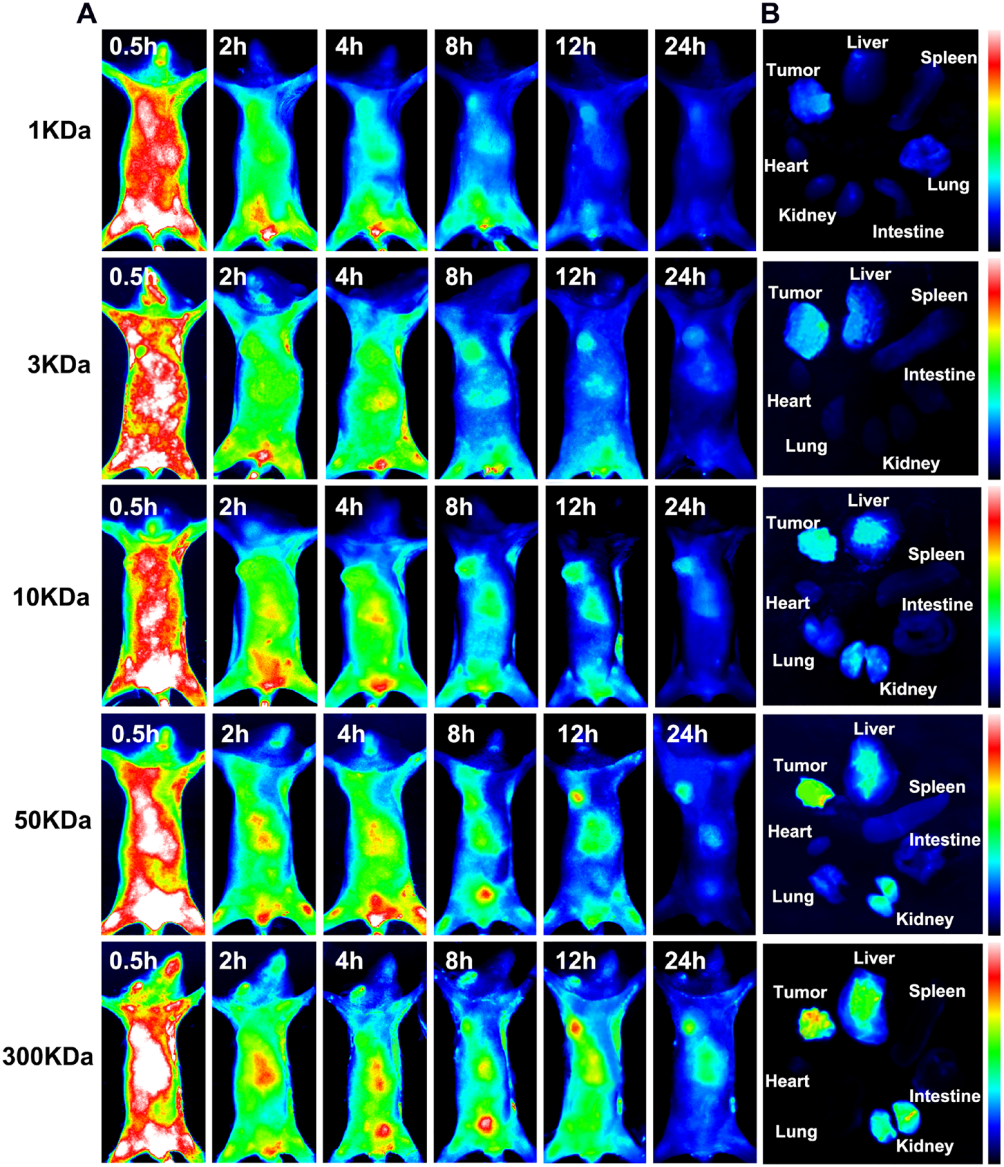
*In vivo* distribution of NIR dye MPA labeled OC (MPA-OC) with different molecular weights in MCF7 tumor-bearing mice **(A)** Live *in vivo* fluorescence imaging of MCF7 tumor- bearing mice at different time points (0.5 h, 2 h, 4 h, 8 h, 12 h, 24 h) post-injection with MPA-OC. **(B)** Fluorescence imaging of tumor and major organs (heart, liver, spleen, lung, kidney and intestine) at 12 h post- injection pf MPA-OC.

To further explore the *in vivo* bio-distribution behavior of OC with different molecular weights, MPA, the derivative of near infrared fluorescence dye Indocyanine Green, was conjugated to OC (MPA-OC) taking advantage of the excellent tissue penetration ability of NIR light. Mice bearing xenograft tumor were intravenously injected with MPA-OC (1KDa, 3KDa, 10KDa, 50KDa and 300KDa) and the dynamic distributions of the nanocarriers were observed for 24 hours and compared (Figure 3). It was found that OC with larger molecular weights had obviously longer *in vivo* retention time, as indicated by stronger *in vivo* fluorescence at each point. Larger nanocarriers also showed stronger tumor signal. At 24 h, nanocarriers with molecular weight higher than 10KDa still demonstrated eminent fluorescence at tumor site, as compared to the negligible signals in the 3 and 1KDa groups. The Tumor/Normal tissue (T/N) ratio of *in vivo* fluorescence imaging was displayed in Supplementary Figure 6. The tumors and organs harvested from the mice after 24 hours confirmed our observation. The larger nanocarriers tended to retain longer in not only tumor, but others normal tissue like liver and kidney. Interestingly, the prominent tumor uptake seemed contradictive to the cellular uptake data, which inspired us to continue in-depth study of the mechanism. We attributed the phenomenon to the fact that macromolecules with higher molecular weight had longer circulation time [27], rendering the nanocarriers more sustained and durable for extravasation and intratumoral accumulation [28]. On the other hand, the accumulation of nanocarriers in tumor area is a result of the extravasation to tumor and clearance from tumor [29]. It has been reported that macromolecule of larger particle size tended to have decreased permeation ability [30], in good parallel with our results. Lower molecular weight OC with higher permeation ability can rapidly enter tumor site, but at the same time, they are more prone to clearance from vasculature, which accounts for the lower degree of tumor accumulation. In contrast, particles with larger molecular weight are accompanied by larger diameters, which may slow the migration rate through the interstitial space, an advantage to allow for greater intratumoral accumulation. However, larger-size particles might mainly accumulate in the perivascular region without reaching a sufficient depth into tumor tissues for effective therapy [31].

To investigate the relationship between tumor size and *in vivo* distribution of OC (10KDa), the tumor models with different tumor diameters (3, 5 and 10 mm) were intravenously injected with 10KDa OC. As shown in Supplementary Figure 7, the larger tumor size model showed the brighter fluorescence signal in tumor, while the fluorescence intensity declined with the reduction of tumor size. Although tumor size indeed impacted on the tumor accumulation efficacy of nanocarriers, all the groups showed obvious tumor accumulation starting from 8 h.

### Tumor penetration of OC with different molecular weights

**Figure 4:**
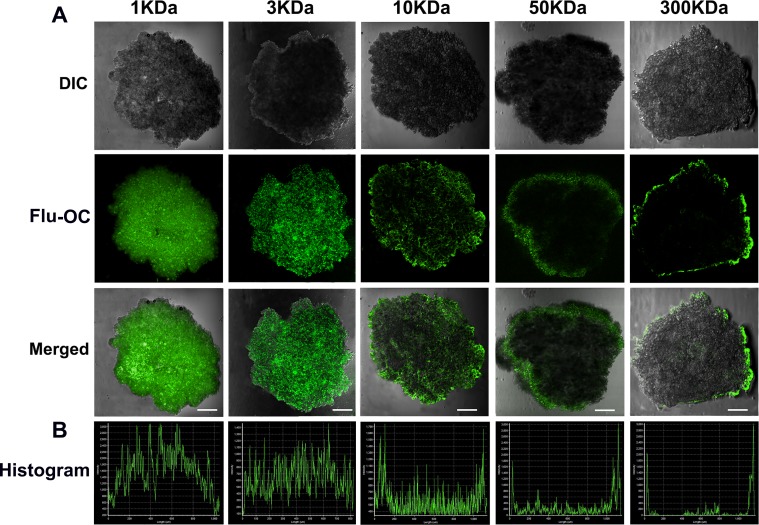
*In vitro* penetration of different molecular weight Flu-OC into the 3D multicellular MCF7 tumor spheroids **(A)** Laser confocal fluorescence images of tumor spheroids after incubation with Flu-OC for 8 h. **(B)** Fluorescence intensity profiles of Flu-OC incubated tumor spheroids at central axis. The length of the scale bars is 150 μm.

To verify the deduction above, a 3D tumor spheroids model was developed to investigate the influence of molecular weight on the permeation ability of the nanocarriers. This spheroids model features similar characteristics with solid tumors such as hypoxia, lower pH and higher inner space [32], but has no tumor vasculature, making it easier to directly observe the penetration depth of the nanocarriers. As shown in Figure 4A, 1KDa OC penetrated into the tumor spheroids to the largest depth, as indicated by the evenly distributed fluorescent signal. The increase of molecular weight caused a gradual decline of the permeation depth of the nanocarriers. While the 3KDa OC could still penetrate into the center of tumor spheroid, the fluorescence in the core region was reduced if the molecular weight increased to 10KDa. Of note, when the molecular weight reached 50 and 300KDa, the fluorescence was mainly detected in the periphery of the spheroids. These findings firmly demonstrated that tumor permeation decreased with larger molecular weight. The semi-quantitative histogram analysis was further carried out to confirm the above observation (Figure 4B). As indicated by the signal intensity at each depth, lower molecular weight OC spread more deeply from superficial to center area, a sharp contrast to the 50 and 300KDa OC with significantly reduced signal at the center. This observation seemed contradictive to the *in vivo* distribution study, in which tumor accumulation of the nanocarriers enhanced with elevated molecular weight. Both tumor accumulation and penetration could influence the drug delivery efficiency of nanocarriers, and further the treatment efficiency. Although the larger molecular weight OC displayed outstanding tumor accumulation ability, their poor penetration made the nanocarriers localize in the perivascular space with little penetration into the inner region of the tumor mass. Meanwhile, the smaller molecular weight OC with excellent penetration displayed relatively weak tumor accumulation. Therefore, the 10KDa OC was the optimal molecular weight with ideal tumor accumulation and penetration depth.

### Drug loading capacity and *in vitro* release profile of OC with different molecular weights

**Figure 5:**
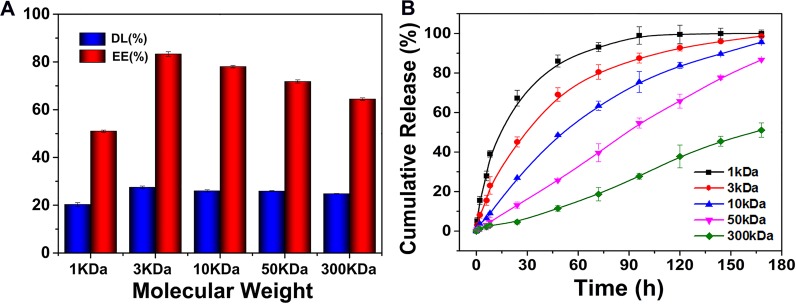
**(A)** The DOX loading capacity and encapsulation efficiency of OC with different molecular weights. **(B)** The release profiles of DOX from OC with different molecular weights at 37°C.

The therapeutic efficacy could be improved by controlling the property of nanocarriers to deliver a greater drug dose to the tumor, which could be achieved through enhancing the payload of the nanocarriers and optimizing the drug release profile. Doxorubicin (DOX) was encapsulated in the core of OC (DOX/OC) as the model drug, which was well dispersed in different media (Supplementary Figure 1B). The drug loading capacity (DL) and encapsulation efficiency (EE) were the highest in the 3KDa group, but gradually decreased with the increase of molecular weight. Generally, when the molecular weights of chitosan were 3KDa, 10KDa and 50KDa, OC had an encapsulation efficiency for DOX greater than 70% (Figure 5A). The *in vitro* release behavior of DOX from OC with molecular weights ranging from 1KDa to 300KDa were compared in PBS. As shown in Figure 5B, different molecular weight nanocarriers displayed varied release rate of the loaded DOX. We observed that 1KDa OC had the fastest release rate, with a large proportion of drug released within 12 hours. Together with the *in vivo* distribution in Figure 3, the nanocarriers existed in circulation until 12 h post injection. The leakage of DOX would result in the toxicity to normal tissues. On the other hand, the premature drug release might lead to the reduced drug concentration released in tumor and unsatisfactory therapeutic efficacy subsequently. In contrast, the 300KDa OC exhibited a slow release with cumulative release less than 50% within 7 days, which led to insufficient drug release. Both 1 and 300KDa OC were inappropriate for drug delivery, while the intermediate molecular weight OC demonstrated a modest release behavior, which increased the drug concentration in tumor tissues and decreased leakage in circulation at the same time. Additionally, we investigate the drug release characteristics of OC in PBS (pH 6.0, tumor microenvironment) and PBS (pH 4.5, endosomal vesicles), respectively. As shown in Supplementary Figure 8, the release rates of DOX in all OC groups were increased with the enhanced acidic environment, indicating the faster release behavior of OC nanocarriers could be existed in tumor tissue or cells.

### Release behavior of DOX/Flu-OC with different molecular weights in tumor cells

**Figure 6:**
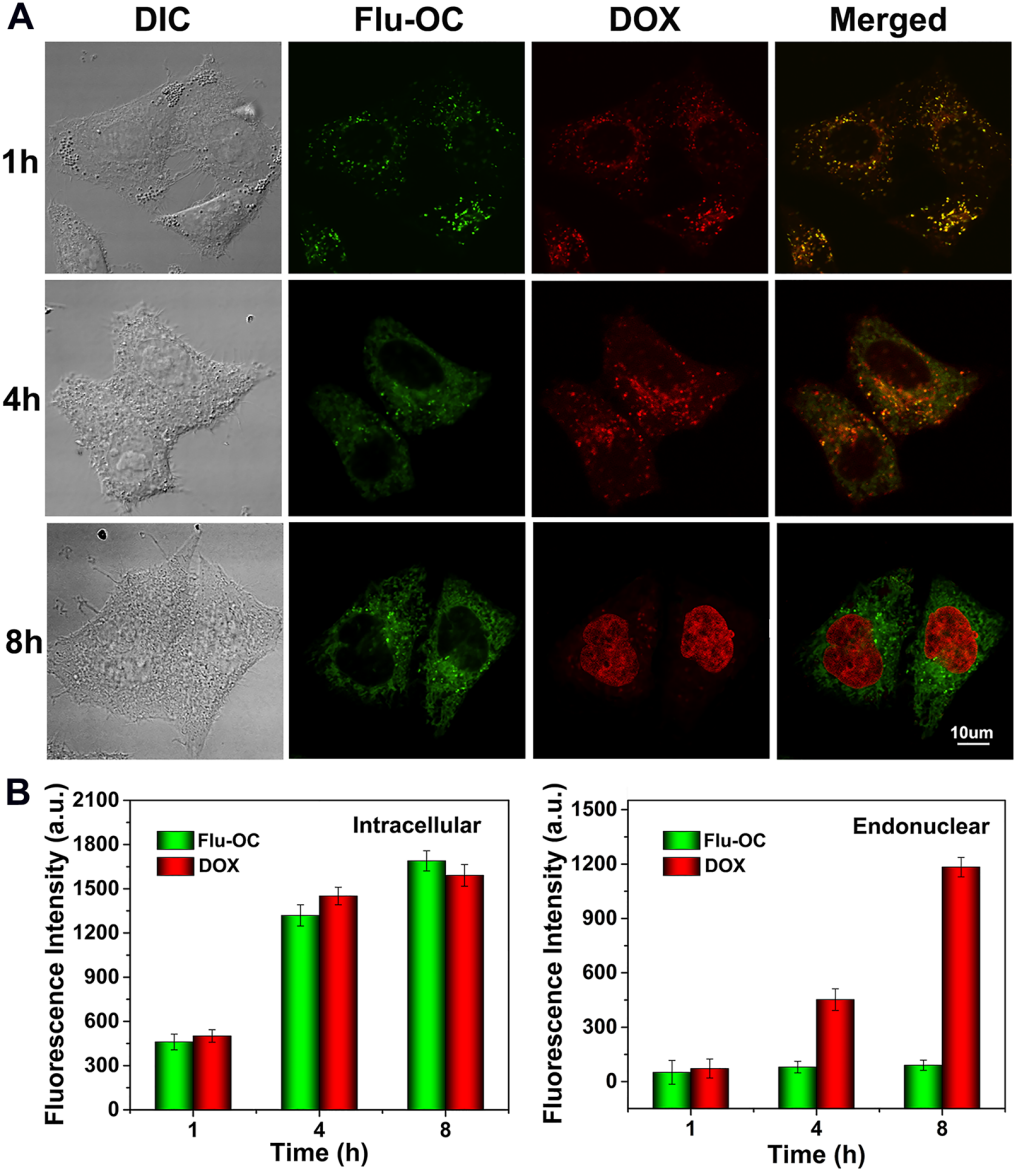
Time dependent endocytosis and drug release of DOX (red) loaded Flu-OC (green) with different molecular weights (DOX/Flu-OC) in MCF7 cells **(A)** Laser confocal fluorescence images of MCF7 cells after incubation with DOX/Flu-OC of different molecular weights for 1, 4 and 8 h. **(B)** Semi-quantification of the fluorescence intensity of Flu-OC and DOX in MCF7 cells.

We then investigated the release of DOX from nanocarriers of 1, 3, 10, 50 and 300KDa in MCF7 cells (Figure 6). The green fluorescence of fluorescein indicated the localization of the carriers, while red signal represented the cellular distribution of DOX. For 10KDa Flu-OC, gradually increased signal of Flu was observed during the first 4h incubation with little change at 8h, indicating cellular uptake the carriers peaked at 4 h. The DOX loaded inside the nanocarriers was co-localized with Flu-OC at 1h, as DOX was not released at this time point. At 4h, the green and red fluorescence began to be separated, with weak red signal noted in the cell nuclei area, implying DOX began to accumulate into nucleus after release from nanocarriers. The almost complete dissociation between DOX and Flu-OC was found at 8h, as indicated by the localization of DOX in cell nucleus and Flu still residing in the plasma. Figure 6B and 6C quantified the fluorescence of DOX and Flu-OC in whole cell and cell nucleus respectively. Consistent with the confocal imaging, gradual increased fluorescent intensity of DOX was observed in nuclear region during the 8h incubation. We also traced the intracellular fate of nanocarriers of other molecular weights and the routing of DOX to cell nucleus (Supplementary Figure 9). The cells took up nanocarriers most efficiently when the molecular was 1KDa. Larger molecular weights resulted in delayed rate of internalization. Obvious fluorescence decline of both DOX and Flu-OC was observed in cells treated with DOX/Flu-OC of 50 and 300KDa, which was in good agreement with Figure 5. DOX release after cellular uptake was time-dependent in all groups with brighter red signal at 8h, but in different efficiencies for various molecular weights. The larger nanocarriers were not only sluggish to enter cells, but also released DOX in a lower rate. At 8h, DOX fluorescence in cell nucleus was much reduced in cells taking up 50 and 300KDa DOX/Flu-OC, but was not obviously changed for other carriers with lower molecular weights. Collectively, these results suggested that 10KDa OC might be optimal as a drug carrier among those of different molecular weights selected.

### Distribution of DOX/Flu-OC with different molecular in tumors

In order to investigae the influence of molecular weight on drug uptake in tumor tissue, tumor bearing mice were sacrificed at 4 and 12 hours post injection. The tumor was sliced for fluorescence imaging. As shown in Supplementary Figure 10, the Flu and DOX fluorescence signals of smaller molecular weight particles (1 KDa and 3 KDa) rapidly appeared in the tumor at 4 h post injection, but rapidly disappeared from tumor tissue at 12 h. As to larger molecular weight particles (50 KDa and 300 KDa), the particles gradually accumulated in the tumor tissues until 12 h post injection, but the fluorescence signal of anticancer drug DOX can only be observed in the superficial area of tumor tissues, indicating the insufficient tumor penetration ability. The intermediate molecular weight particle (10 KDa) demonstrated both persistent tumor accumulation and evenly DOX distribution in tumor tissue, which could further confirm the outstanding therapeutic efficacy of 10 KDa OC.

### Therapeutic efficacy of DOX/OC with different molecular weights

**Figure 7:**
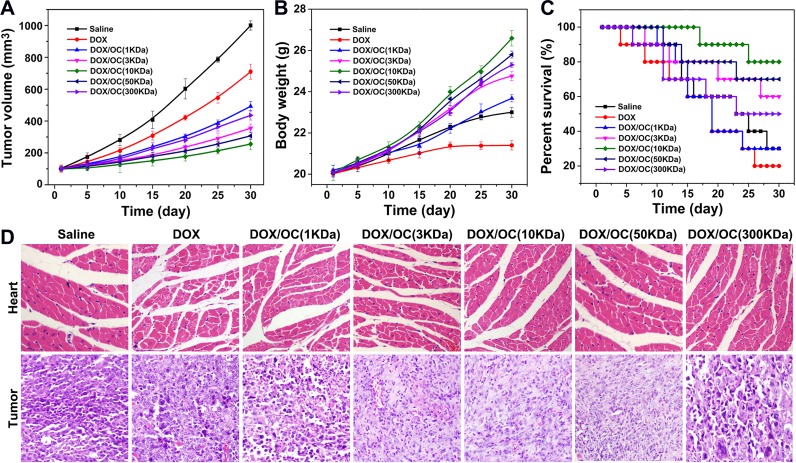
*In vivo* antitumor efficacy of DOX/OC with different molecular weights **(A)** Curves of tumor growth in response to different treatments. **(B)** Changes of the body weights in mice during different treatments. **(C)** Survival rates of MCF7 tumor bearing mice after different treatment over time. **(D)** Histological analysis of tumor and heart sections collected from different treatment groups.

Finally, the *in vivo* anti-tumor efficacy and toxicity of the drug loaded carriers of different molecular weights were compared (Figure 7) in MCF-7 tumor bearing mice. Tumor volume, body weight and survival rate were monitored during the 30 days’ treatment. Compared with the saline control, the most significant delay of tumor growth was found in mice treated with 10KDa OC loaded with DOX. This might be explained by the superiority of 10KDa OC in cellular uptake, tumor penetration and drug release efficiency. Other molecular weights nanocarriers demonstrated tumor inhibition to lesser extents, but still higher than director systemic injection of DOX, as a result of non-specific distribution of free DOX. Lower MW particles (1 and 3 KDa) displayed a lesser extents of tumor inhibition than 10 KDa, which was mostly caused by the relatively low tumor accumulation (Figure 3). While the low therapeutic efficacy of large molecular weight OC was mostly attributed to the poor tumor penetration. DOX caused a substantial weight loss in mice, as a result of severe systemic toxicity. In sharp contrast, the weights of mice treated with nanodrugs increased with the most weight gaining in 10KDa group. The potent tumor inhibition efficacy and low toxicity of 10KDa DOX/OC were translated into prolonged animal survival (Figure 7C). The tumor inhibition and cardiotoxicity of the different carriers were further confirmed by H&E staining of tumor and normal tissues (Figure 7D). Enhanced tumor killing effects were achieved in mice treated with the DOX loaded nanocarriers than those with free DOX. Also, 10KDa DOX/OC exhibited the most pronounced anti-tumor effects compared with other molecular weights. Of note, 1 and 300KDa DOX/OC only induced marginal tumor necrosis; it was possibly because 1KDa DOX/OC were cleared out from the body before drug release into tumor site, while 300KDa DOX/OC could not effectively penetrate into tumor. Cardiotoxicity was only observed in mice treated with free DOX or 1KDa DOX/OC, which could also be explained by the fast drug release from 1KDa OC into blood rather than tumor. To further investigate the toxicity of DOX/OC with different molecular weights, the acute toxicity was studied using nude mice. After 7 days post injection of DOX/OC, the blood was collected and serum bio-chemical parameters were examined. As shown in Supplementary Figure 11, the aspartate aminotransferase (AST), alanine aminotransferase (ALT) and blood urea nitrogen (BUN) values elevated with increased molecular weight, demonstrating stronger liver and kidney toxicity of the higher molecular weight DOX/OC. Also, the CK value of free DOX and 1 KDa DOX/OC treated mice were much higher than that of 3-300 KDa DOX/OC, which might be caused by the leakage of DOX from 1 KDa DOX/OC in blood circulation and the nonselective distribution of DOX.

## MATERIALS AND METHODS

### Materials

Chitosan with average molecular weight (MW) of 1KDa, 3KDa, 10KDa, 50KDa and 300KDa was purchased from Aoxing Biotechnology Co. Ltd.. Doxorubicin (Dox), fluorescein (Flu) were purchased from Sigma-Aldrich. RPMI-1640 medium, thiazolyl blue (MTT), fetal bovine serum and trypsin were purchased from Keygen Biotech. Inc.. Octanal, sodium borohydride (NaBH4) were purchased from China National Medicine Corporation Ltd.. MPA (MW: 995) was prepared in our laboratory following Achilefu's report [33, 34]. All other solvents and reagents used in this study were certified analytical reagent grade.

### Synthesis of OC

Chitosan (1 g) of different molecular weight was dissolved in 20 mL succinic acid (4.8% w/v) and drop added octanal with stirring. After 4 h reaction, the solution was adjusted to PH4.5 using NaOH, then added NaBH4 and stirred for 12 h. The product was purified by dialyzing against distilled water and followed by lyophilization. Octyl-chitosan (1g) with different molecular weight was dissolved in 50 mL phosphate buffer saline (PBS) solution, then treated with a probe-type sonicator for 10 min at 200W. The nanocarriers were formed by self-assembly of the amphiphilic octyl-chitosan.

### Characterization of OC

The size distribution and the stability of the nanocarriers was measured by dynamic light scattering (DLS, LPSA, Malvern Instruments, UK). The morphology of OC was characterized by transmission electron microscopy (TEM, Philips FEI Tecnai G220s-TWIN, Netherlands). A drop of OC solution was added on the surface of the gird, and then phosphotungstic acid was used to negative staining before microscope observation. The zeta-potential of the nanocarriers was measured by Zetasizer (Brookhaven Instruments Corporation, USA).

### Toxicity evaluation of OC

MTT assay was conducted to evaluate the cytotoxicity of OC with different molecular weights in normal cell lines L02 (liver cells) and HUVEC (human umbilical vein endothelial cells). The cells were seeded on 96-well plates with a density of 5×10^3^ cells/well and incubated for 12 h under CO_2_ environment. In consideration of the *in vivo* therapeutic doses of DOX and the drug loading efficacy, the doses of OC was calculated as a range of 0.26 mg - 0.4 mg in each mouse (20 g). Generally, there are ∼2 mL of blood in each mouse, the concentrations of OC in the blood were calculated as 0.13 mg/mL – 0.2 mg/mL. Therefore, 0.125 mg/mL was chosen as the lowest administration dose. For larger dose might induce toxicity, 2 mg/mL (ten times of 0.20 mg/mL) was chosen as the highest concentration. After treatment with OC at a concentration range of 0.125 mg/mL - 2 mg/mL, the cells were further incubated for another 48 h. MTT solution (20 uL, 5.0 mg/mL) was added into each well and incubated with the cells for 4 h. Then, the media containing MTT was carefully removed and 150 uL dimethyl sulfoxide was added into each well for dissolve the formazan crystal. The plates were shaken for 10 min before measured the optical density at 560 nm. (n=6)

In order to investigate the *in vivo* toxicity, nude mice with half males and half females was used in the experiment. Those mice were treated in accordance with the Guidance Suggestions for the Care and Use of Laboratory Animals. Six-to-seven weeks old nude mice were maintained under aseptic conditions in a small animal isolator and were housed in standard cages with free access to food and water. The nanocarriers with different molecular weights were injected through the tail vein, with saline treated group as control. As large dose is more applicable to induce toxic reaction in animals. So, we choose 5 times dose of OC as high as the OC used in the therapeutic experiment for *in vivo* toxicity. The concentration of OC was calculated as 98.15 mg/kg for 1KDa, 65.9 mg/kg for 3KDa, 71.15 mg/kg for 10KDa, 71.55 mg/kg for 50KDa and 75.80 mg/kg for 100KDa. The body weight of each mouse was monitored every five days over 30 days. To further investigate the toxicity of the nanocarriers with different molecular weight, the organs were collected and weighed after 30 days’ treatment. The isolated organs were sliced to 8 mm and stained with Hematoxylin and Eosin (H&E) for further observation by Olympus optical microscope. At the same time, the blood of the nanocarriers treated mice was collected for access of biochemical and hematological parameters.

### Cellular uptake of OC

Fluorescein was conjugated to OC (Flu-OC) through amidation reaction for cellular uptake evaluation. MCF7 breast cancer cells were seeded in laser scanning confocal microscope (LSCM, FV1000, Olympus, Japan) culture dishes (5×10^5^ cells per well) and incubated with Flu-OC with different molecular weights respectively for 4 h. Hoechst solution (100uL, 10ug/mL) was used to stain nucleus. After washed with PBS for 3 times, the cells were imaged by LSCM. For further evaluation, MCF7 cells were incubated with Flu-OC of different molecular weights and collected after incubated of different time points. After washed with PBS for 3 times, the cells were lysed and evaluated the fluorescence signal using a Lambda 35 UV-Vis spectrophotometer (Perkin-Elmer, USA). The area under the Flu-OC concentration- time curve (AUC) was calculated by the linear trapezoidal method.

Further experiment was conducted to evaluate the mechanism of cellular uptake. MCF7 breast cancer cells were seeded in laser scanning confocal microscope (LSCM, FV1000, Olympus, Japan) culture dishes (5×10^5^ cells per well) and incubated with Flu-OC with different molecular weights after pre-incubation with β-cyclodextrin and sucrose, respectively. After washed with PBS for 3 times, the cells were imaged by LSCM.

### *In vivo* distribution of OC

All the animal experiments in this study were conducted under the Animal Management Rules of the Ministry of Health of the People's Republic of China and the guidelines for the Care and Use of Laboratory Animals of China Pharmaceutical University. Mice bearing xenograft tumor was established as followed: female nude mice were subcutaneously inoculated with 1×10^6^ MCF7 cells into the left axillary fossa. When the diameter of tumors reached 5 mm, the mice were used for fluorescence imaging and therapeutic experiment. The mice were intravenous injected with MPA labeled OC (MPA-OC) with different molecular weights separately. The mice were imaged using the NIR fluorescence imaging system at different time points. The mice were sacrificed after 24 hours post injection. The harvested tissues were imaged using the NIR fluorescence imaging system.

Tumor models were established as followed: MCF7 cells (1×10^6^) were injected into the left axillary fossa of each nude mice. After the tumor diameter reached about 3, 5, 10 mm, the mice were intravenous injected with MPA labeled OC (MPA-OC) with different molecular weights separately. The mice were imaged using the NIR fluorescence imaging system at different time points.

### Tumor spheroid penetration ability evaluation of OC

The 3D tumor spheroids of MCF7 cells were developed according to the previous report [35]. Each well of 96-well plates was coated with a layer of sterilized agarose-based DMEM (2% w/v). MCF7 cells were seeded into each well at a density of 1 ×10^5^ cells per well in the complete medium. Then, the plates were gently shaken for 5 min and cultured at 37 degrees centigrade with the presence of 5% CO_2_. The tumor spheroids were allowed to grow for 7 days to attain a diameter about 1000 um. The uniform and compact tumor spheroids were selected for the following studies.

The culture of 3D tumor spheroids was different from that of solid tumors. The 3D tumor spheroids were cultured in 96-well plates containing Flu-OC for fluorescence imaging. However, for *in vivo* imaging, Flu-OC were injected through tail intravenous, and accumulated into solid tumors through Enhanced Permeation Retention (EPR) effect. And the diameter of tumor spheroids and the solid tumors was different, therefore, the treated time of these two models was also different. The tumor spheroids were incubated with Flu-OC with different molecular weights for 8 h. Subsequently, the tumor spheroids were washed with PBS and imaged using LSCM. The fluorescence intensity profiles of tumor spheroids were measured by ROI analysis using Olympus FV1000.

### Characterization of DOX/ OC

Anti-cancer agent doxorubicin (DOX) was encapsuled in the core of OC as a model drug. In order to determine the entrapment efficiency, the drug loaded OC solutions were lyophilized and then dissolved in DMSO. The absorbance peaks (227 nm for PTX and 430 nm for Su) of the solutions were determined, and the quantities of the loaded drugs were calculated according to the standard curve (PTX: y=0.0292x-0.0001, R^2^=0.9986; Su: y=0.0051x-0.0023, R^2^=0.9987). The entrapment efficiency (EE) and drug loading content (DL) were calculated according to the following equations: DL= (mass of drug loaded/ mass of drug loaded and nanocarriers) × 100%; EE= (mass of drug loaded/ mass of drug fed) × 100%. The *in vitro* release profile of DOX from different molecular weight OC were investigated by a dialysis method. DOX/OC with different molecular weights were dialyzed against PBS with gentle shaking. At every time point, 3 mL dialysate was withdrawn and replaced with the same volume of fresh PBS (pH7.4, pH6.0 and pH4.5). The cumulative drug concentration in the dialysate of each time point was measured using the UV-Vis spectrophotometer. The stability of OC and DOX/ OC was measured by respectively adding Glutathione (GSH, 5%), NaCl (0.9%), serum (10%) and DMEM, and observed after 12 hours.

### The distribution of DOX/ Flu-OC in tumor cells

DOX/ Flu-OC with different molecular weight was incubated with MCF7 cells laser scanning confocal microscope culture dishes for 1h,4h and 8h. The fluorescence signals of DOX and Flu-OC were detected using LSCM to evaluate the drug release in tumor cells. The intensity of two fluorescent signals in cells and nuclear was analyzed using Olympus FV1000.

### The distribution of DOX/ Flu-OC in tumor tissues

To assess the *in vivo* distribution of different molecular weight OC nanocarriers in tumor tissues, the tumor-bearing mice were randomly divided into 5 groups. Each group was intravenously injected with 1 KDa, 3 KDa, 10 KDa, 50 KDa and 300 KDa DOX loaded OC nanocarriers. After 4 and 12 hours post injection, tumor-bearing mice were sacrificed and the tumor tissues were collected, frozen and sliced for fluorescence imaging.

### Therapeutic efficacy of DOX/OC in tumor-bearing mice

The MCF7 tumor-bearing mice were randomly divided into seven groups (n=20) and separately treated with saline, DOX, DOX/OC (1KDa), DOX/OC (3KDa), DOX/OC (10KDa), DOX/OC (50KDa), DOX/OC (300KDa). The group treated with saline was used as control. The other groups maintained a dose of 5 mg/kg equivalent DOX in each mouse. The tumor volume was measured every 5 days. The body weight and the survival rate was also monitored over 30 days. To further investigation, the tumor and heart of the seven groups were excised for pathological analysis.

The acute toxicity of DOX/OC was further evaluated. DOX/OC with different molecular weights were injected through the tail vein, and the blood was collected after 7 days post-injection. Serum bio-chemical parameters, including aspartate aminotransferase (AST, index of hepatotoxicity), alanine aminotransferase (ALT, index of hepatotoxicity), blood urea nitrogen (BUN, index of nephrotoxicity) and creatine kinase (CK, index of cardiotoxicity), were examined.

### Statistical analysis

Significant differences were determined using the Student's t-test where differences were considered significant when p < 0.05. All data are expressed as mean± standard error of the mean.

## CONCLUSIONS

In this work, the macromolecular drug carrier, octyl chitosan, was selected as a typical model to explore the influence of molecular weight on drug delivery efficiency. It was found that molecular weight could impact intratumoral accumulation and penetration, as well as drug loading and releasing profiles of the nanocarriers. These factors together determined drug concentration in tumor and finally the therapeutic efficacy. We demonstrated that 10KDa was the optimal molecular weight for drug delivery over the 1-300KDa range, achieving both effective drug accumulation in tumor and sufficient penetration, which were ultimately translated into better therapeutic efficacy compared with other molecular weights. More importantly, this finding enriched the evidence for optimization of macromolecular drug carriers, shedding light on better design of drug delivery systems.

## SUPPLEMENTARY MATERIALS FIGURES


