# Supplementary material for: Optimizing molecular weight of octyl chitosan as drug carrier for improving tumor therapeutic efficacy
Source: Oncotarget. 2017 Jul 22;8(38):64237–49. doi: 10.18632/oncotarget.19452 (PMC5609998; doi:10.18632/oncotarget.19452)
Supplement: Supplementary file 1 [file oncotarget-08-64237-s001.pdf]

Optimizing molecular weight of octyl chitosan as drug carrier for improving tumor therapeutic efficacy

SUPPLEMENTARY MATERIALS

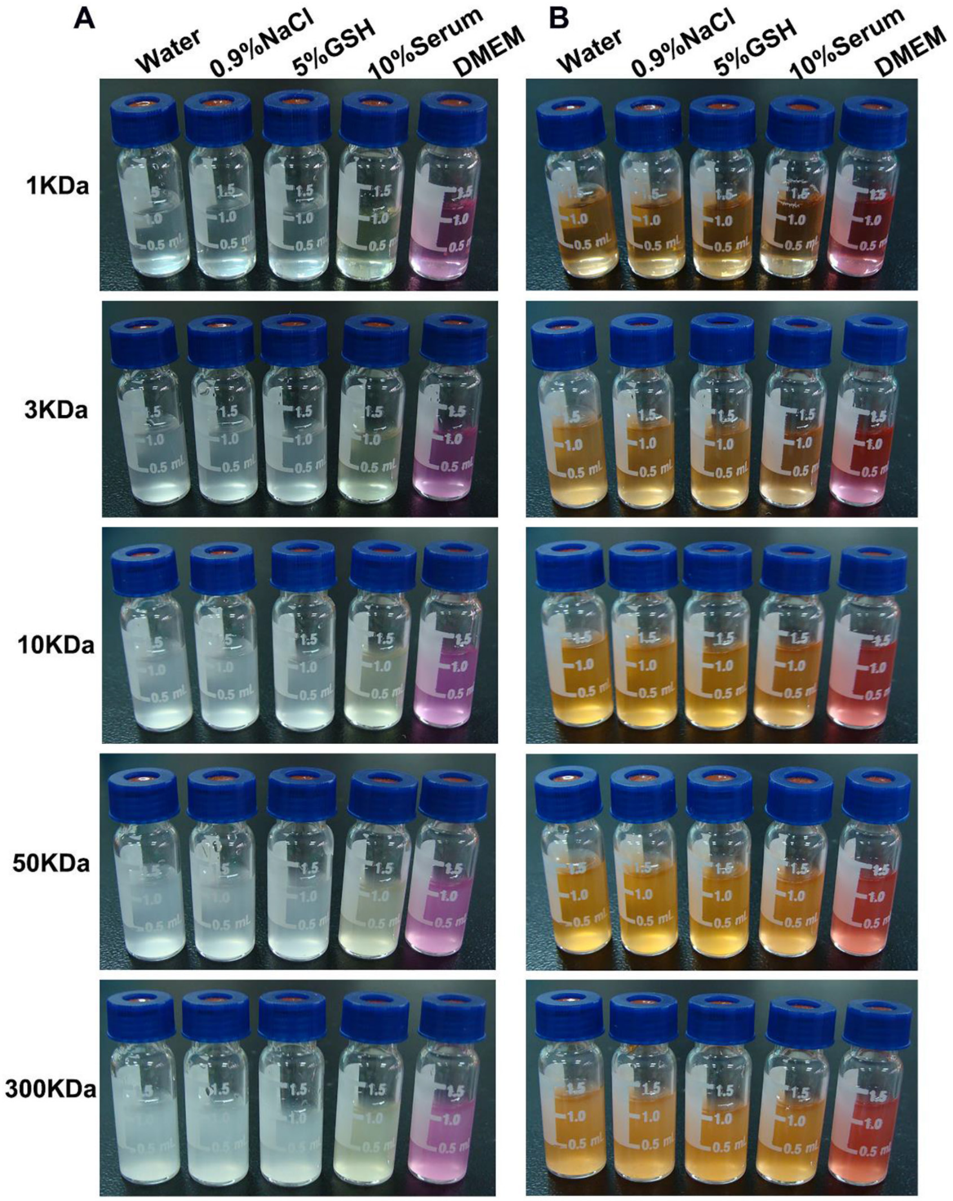

Supplementary Figure 1: The stability of nanoparticles. **(A)** The photos of OC and **(B)** DOX/OC of different molecular weights after treatment with different media.

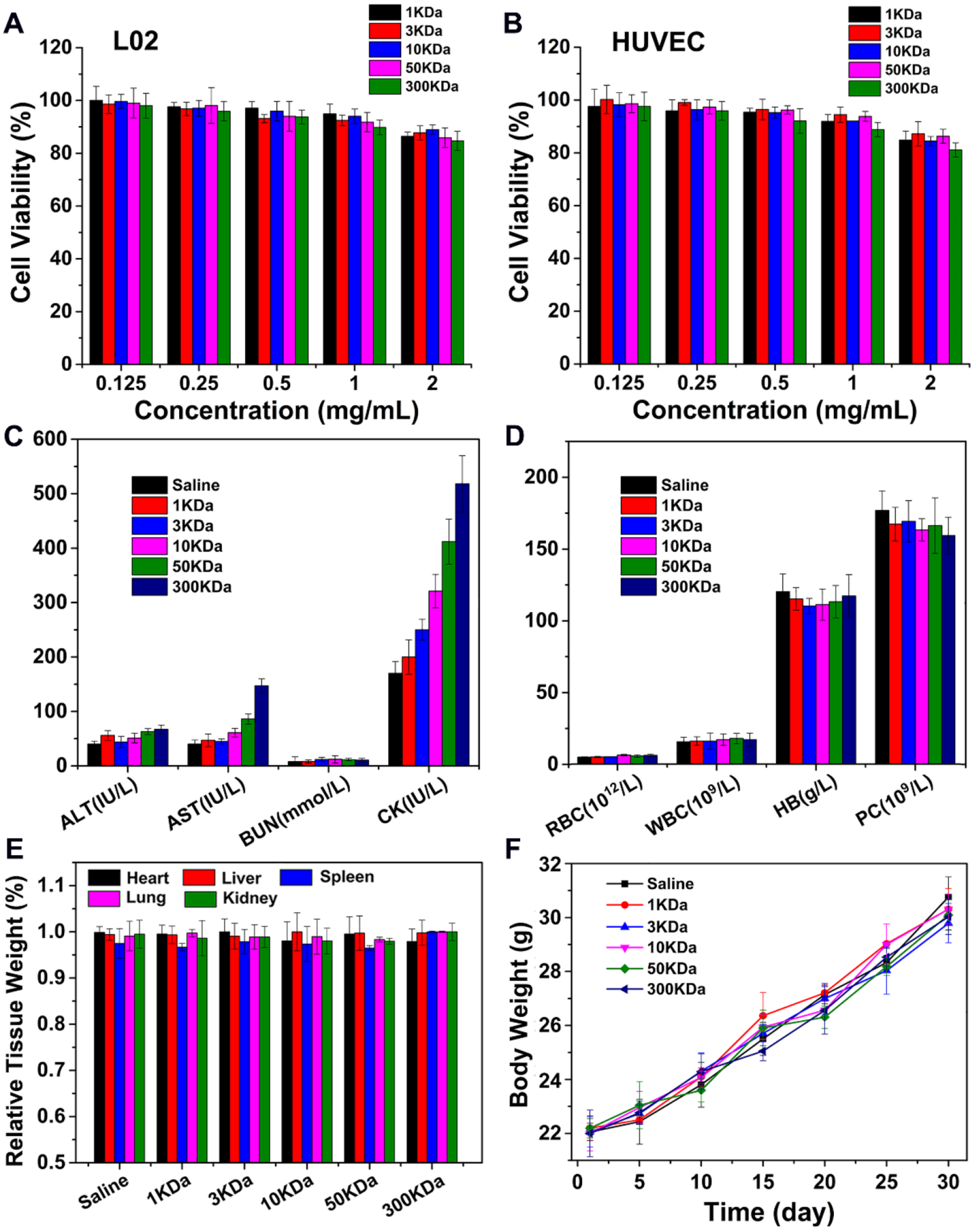

Supplementary Figure 2: The toxicity of OC with different molecular weights. **(A)** MTT assay of L02 and **(B)** HUVEC cells after incubation with OC after 48 h. **(C)** The biochemical and **(D)** hematological parameters were compared after different treatments. **(E)** Weight changes of major organs after different treatments. **(F)** Body weight changes of different treatment groups.

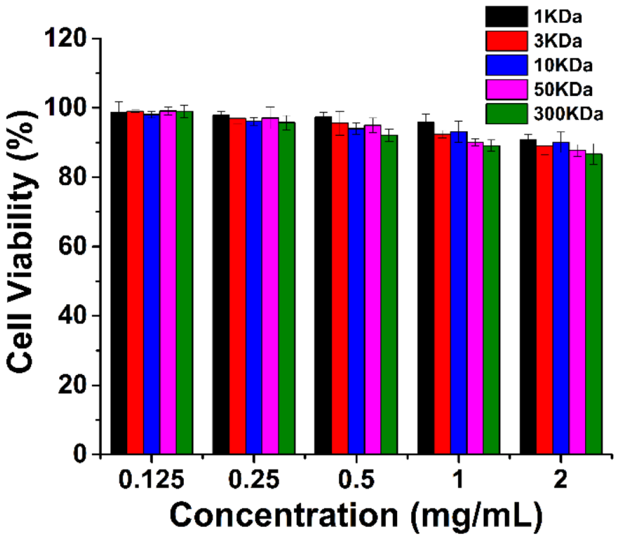

Supplementary Figure 3: The cytotoxicity of OC with different molecular weights in MCF7 cells.

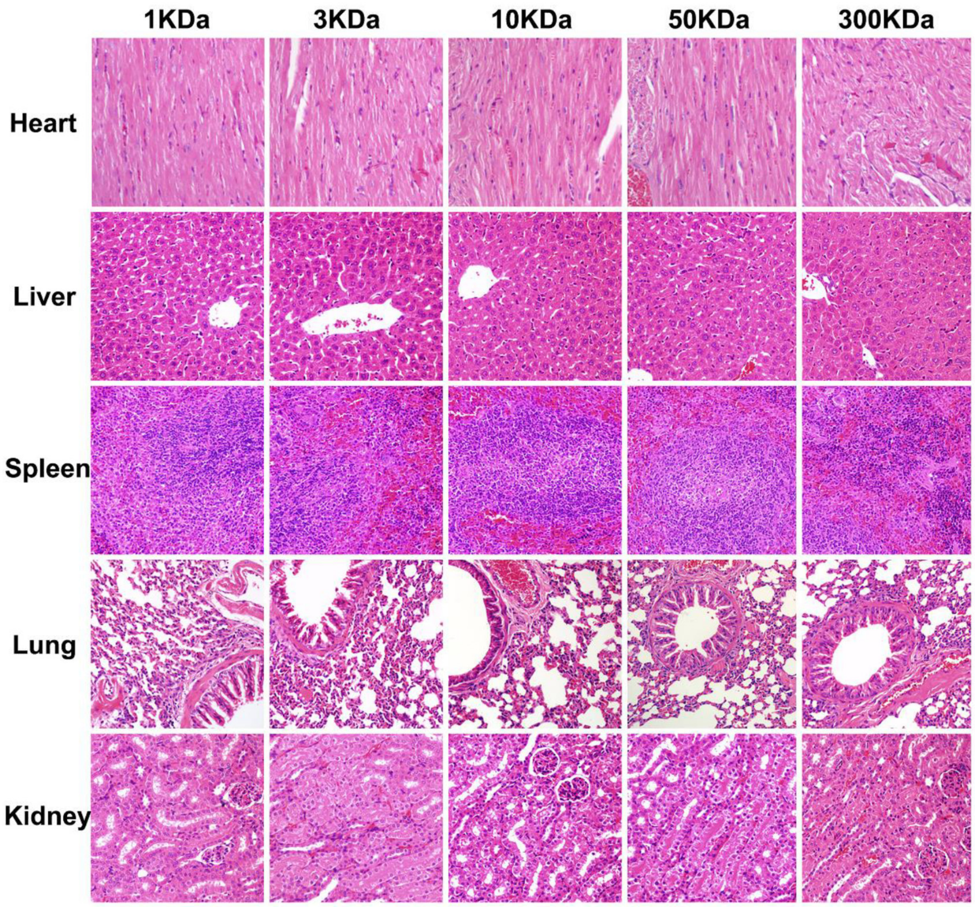

Supplementary Figure 4: Histopathological analysis of the major organs (heart, liver, spleen, lung and kidney) after different treatments.

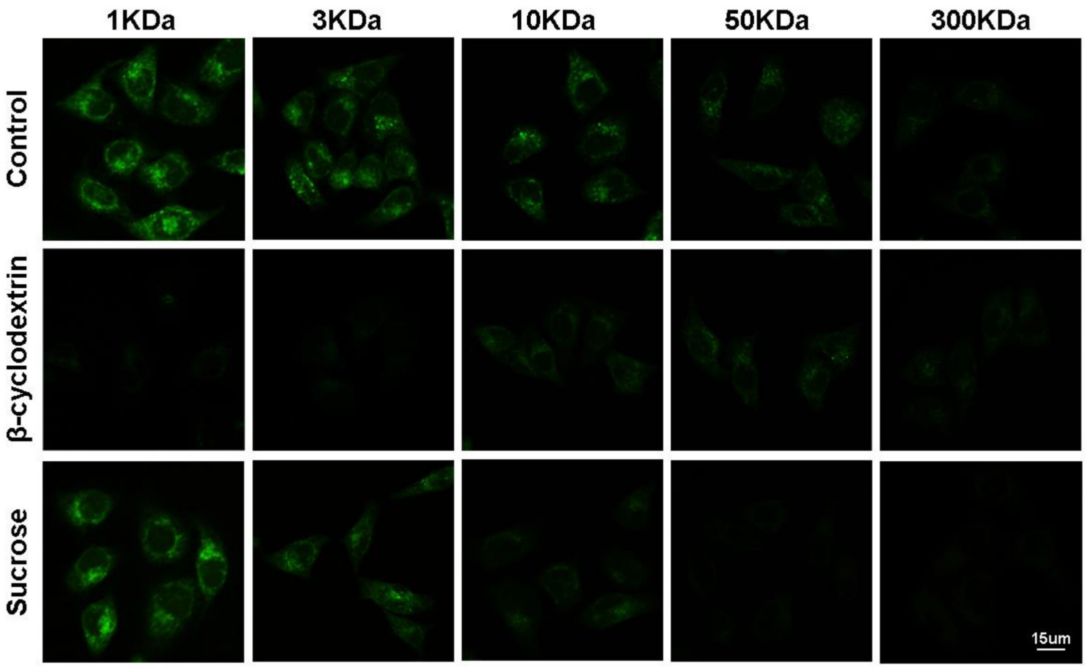

Supplementary Figure 5: Fluorescence images of MCF7 cells incubated with Flu-OC with different molecular weights after pre-incubation with β-cyclodextrin and sucrose, respectively.

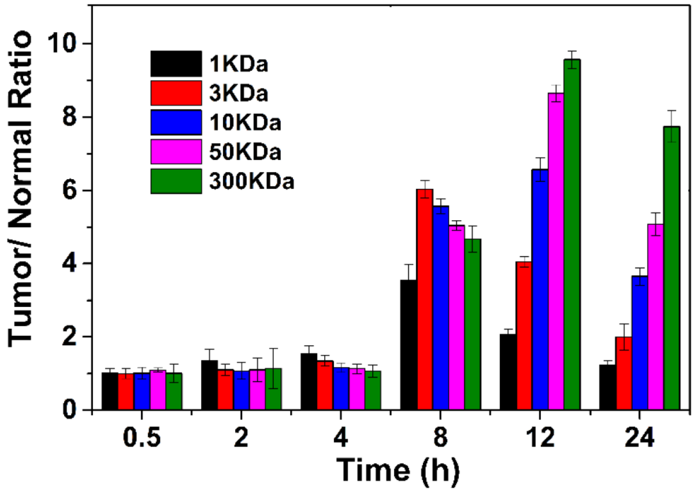

Supplementary Figure 6: Tumor/ normal tissues ratio of *in vivo* image.

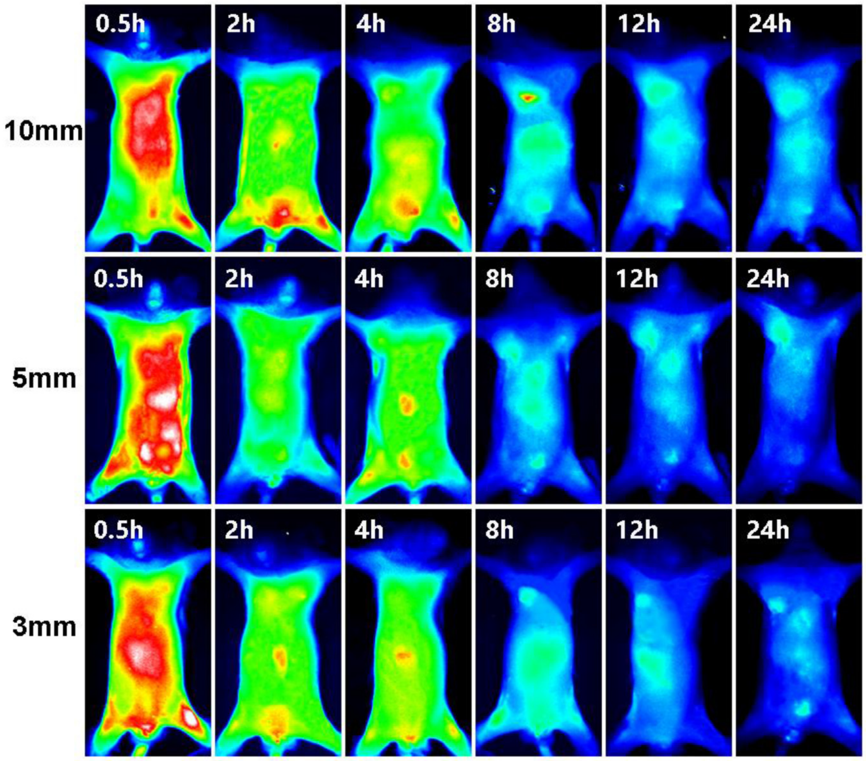

Supplementary Figure 7: ***In vivo*** **fluorescence imaging of NIR dye labeled 10KDa OC at different time point (0.5 h, 2 h, 4 h, 8 h, 12 h, 24 h) in MCF7 tumor bearing mice with different tumor diameter (10mm, 5mm and 3mm).**

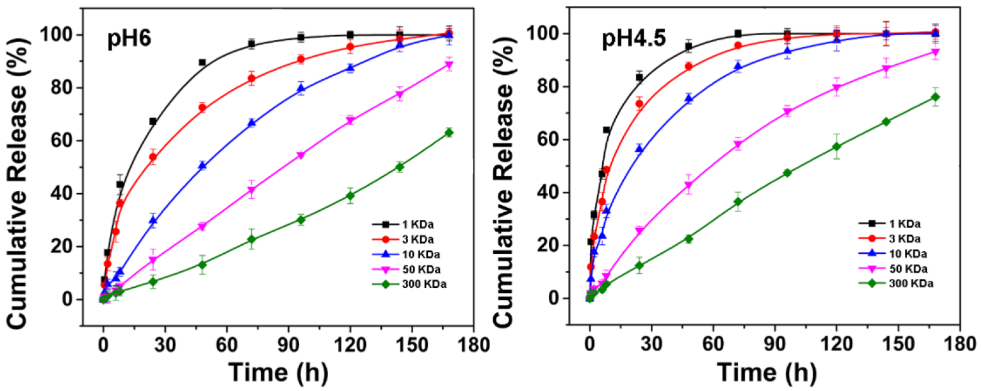

Supplementary Figure 8: The release profiles of DOX from OC with different molecular weights in PBS (pH 6) and PBS (pH 4.5).

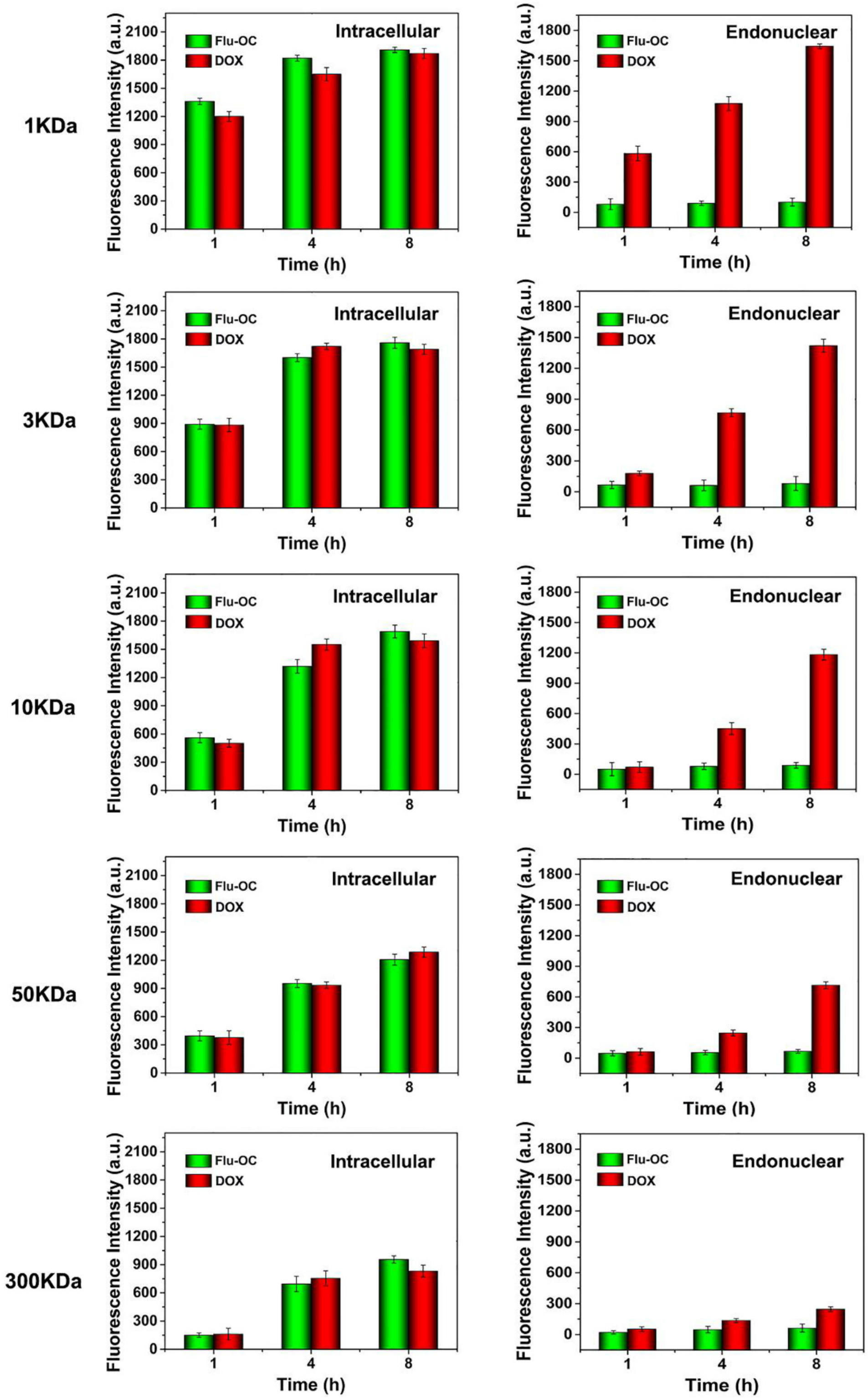

Supplementary Figure 9: Time dependent fluorescence intensity of DOX and Flu-OC with different molecular weights in cells and nuclei.

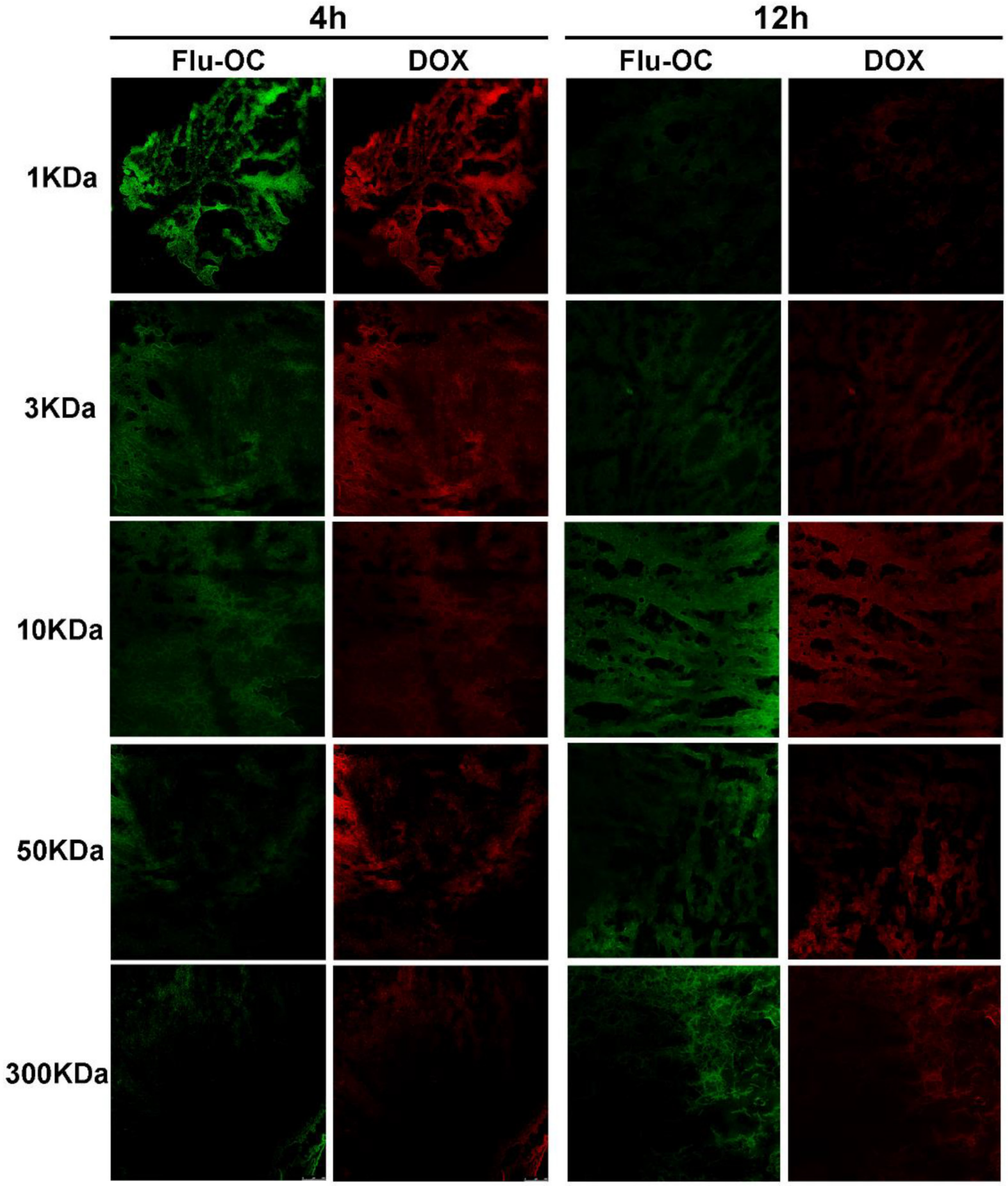

Supplementary Figure 10: Fluorescence imaging of tumor slices after intravenous injection of different molecular weight OC nanocarriers at 4 and 12 hours. Green: FITC; Red: DOX.

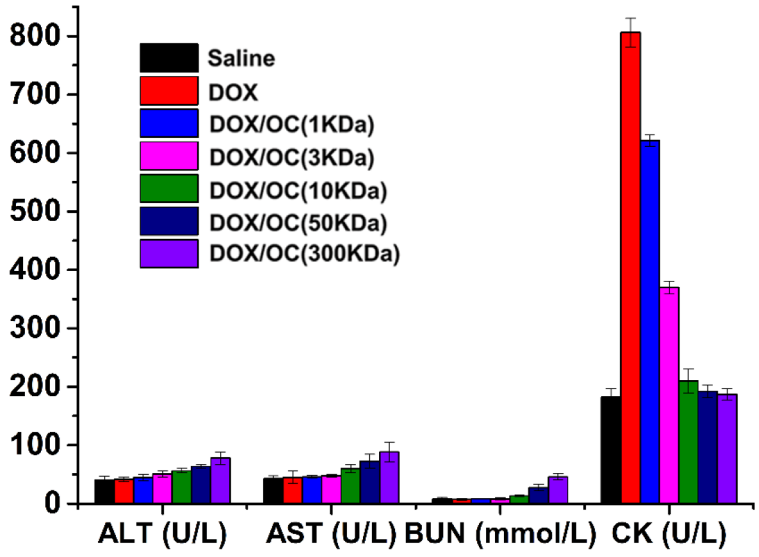

Supplementary Figure 11: Serum biochemical parameters (ALT, AST, BUN and CK) of mice after 7 days post-injection with different molecular weight of DOX-loaded OC nanocarriers.
